# Photocatalytic Protein Damage by Silver Nanoparticles Circumvents Bacterial Stress Response and Multidrug Resistance

**DOI:** 10.1128/mSphere.00175-19

**Published:** 2019-05-01

**Authors:** Tianyuan Shi, Qiuxia Wei, Zhen Wang, Gong Zhang, Xuesong Sun, Qing-Yu He

**Affiliations:** a Key Laboratory of Functional Protein Research of Guangdong Higher Education Institutes, Institute of Life and Health Engineering, College of Life Science and Technology, Jinan University, Guangzhou, China; University of Nebraska Medical Center

**Keywords:** silver nanoparticles, antibiotic resistance, iTRAQ, light, protein aggregation

## Abstract

Silver nanoparticles (AgNPs) are known for their broad-spectrum antibacterial properties, especially against antibiotic-resistant bacteria. However, the bactericidal mechanism of AgNPs remains unclear. In this study, we found that the bactericidal ability of AgNPs is induced by light. In contrast to previous postulates, visible light is unable to trigger silver ion release from AgNPs or to promote AgNPs to induce reactive oxygen species (ROS) in Escherichia coli. In fact, we revealed that light excited AgNPs to induce protein aggregation in a concentration-dependent manner in E. coli, indicating that the bactericidal ability of AgNPs relies on the light-catalyzed oxidation of cellular proteins via direct binding to proteins, which was verified by fluorescence spectra. AgNPs likely absorb the light energy and transfer it to the proteins, leading to the oxidation of proteins and thus promoting the death of the bacteria. Isobaric tags for relative and absolute quantitation (iTRAQ)-based proteomics revealed that the bacteria failed to develop effective resistance to the light-excited AgNPs. This direct physical mechanism is unlikely to be counteracted by any known drug resistance mechanisms of bacteria and therefore may serve as a last resort against drug resistance. This mechanism also provides a practical hint regarding the antimicrobial application of AgNPs—light exposure improves the efficacy of AgNPs.

**IMPORTANCE** Although silver nanoparticles (AgNPs) are well known for their antibacterial properties, the mechanism by which they kill bacterial cells remains a topic of debate. In this study, we uncovered the bactericidal mechanism of AgNPs, which is induced by light. We tested the efficacy of AgNPs against a panel of antimicrobial-resistant pathogens as well as Escherichia coli under conditions of light and darkness and revealed that light excited the AgNPs to promote protein aggregation within the bacterial cells. Our report makes a significant contribution to the literature because this mechanism bypasses microbial drug resistance mechanisms, thus presenting a viable option for the treatment of multidrug-resistant bacteria.

## Abstract (precis)

Although silver nanoparticles (AgNPs) are well known for their antibacterial properties, the mechanism by which they kill bacterial cells remains a topic of debate. In this study, we uncovered the bactericidal mechanism of AgNPs, which is induced by light. We tested the efficacy of AgNPs against a panel of antimicrobial-resistant pathogens as well as Escherichia coli under conditions of light and darkness and revealed that light excited the AgNPs to promote protein aggregation within the bacterial cells. Our report makes a significant contribution to the literature because this mechanism bypasses microbial drug resistance mechanisms, thus presenting a viable option for the treatment of multidrug-resistant bacteria.

## INTRODUCTION

The improper use of antibiotics promotes the development of antibiotic-resistant bacteria (1, 2). The widespread incidence of multidrug-resistant and pan-resistant bacterial infections has become a serious challenge in clinical practice, and the resistance can be transmitted between bacteria through plasmids (3). Unfortunately, resistant bacteria emerge shortly after the clinical use of new artificially synthesized antibiotics (4). Bacteria possess several universal mechanisms to counteract various kinds of antibiotics, including mutation/modification of the effective sites, production of enzymes that specifically degrade antibiotics, alteration of membrane permeability, and tuning of the translation system (5). Therefore, there is an urgent need to develop bactericides with alternative mechanisms.

Silver nanoparticles (AgNPs) have shown effective inhibition of drug-resistant bacteria (6). It appears that bacteria exhibit low resistance to AgNPs (7), supporting their use as a promising bactericide. AgNPs have been reported to inhibit many species of bacteria, including Staphylococcus aureus (8), Pseudomonas aeruginosa, Escherichia coli, Bacillus subtilis, Vibrio cholerae, Salmonella enterica serotype Typhi, Enterococcus faecalis, *Klebsiella* sp., *Listeria* sp., and Acinetobacter sp. (9). In particular, AgNPs are extremely effective in the suppression of multidrug-resistant E. coli MREC33 (10), Micrococcus luteus (11), Klebsiella pneumoniae (11), S. aureus (11), Streptococcus pneumoniae (12), and S.enterica serotype *Typhi* (12).

A previous study showed that AgNPs penetrate bacterial cells (13), indicating that AgNPs can directly interact with cellular macromolecules. However, the bactericidal mechanism of AgNPs is not clear, with several controversial hypotheses as follows. (i) Oxidized AgNPs release free silver ions from the surface of the NPs to exert toxic effects on bacteria (14). However, a surface containing immobilized AgNPs exhibited a better antibacterial effect than one coated with silver ions (15), indicating that AgNPs and Ag^+^ have different bactericidal pathways. (ii) AgNPs disrupt the cell membrane/wall (13, 16) and thus inhibit aerobic respiration (17, 18), damage DNA (8, 19, 20), and perturb protein biosynthesis and folding (21–23). (iii) Reactive oxygen species (ROS) are induced by light-excited AgNPs and then kill the bacteria (24). However, some studies found that AgNPs are antioxidants *in vitro* (25, 26).

In this study, we investigated a novel bactericidal mechanism of AgNPs. This bactericidal mechanism involves direct light-excited protein oxidation catalyzed by the AgNPs, which is not easily counteracted by the known antibiotic resistance mechanisms of bacteria. Indeed, AgNPs can inhibit carbapenem-resistant bacteria containing the *ndm-1* gene. This study may provide insight into effective treatment of drug-resistant bacterial infections.

## RESULTS

### Characterization of AgNP morphology.

The size distribution of AgNPs used in this study was analyzed by dynamic light scattering (DLS). The diameter of the AgNPs was 11.12 ± 0.07 nm, indicating that the AgNPs were uniform. Further transmission electron microscopy (TEM) detection demonstrated that AgNPs were regularly spherical. These results indicated the uniform morphology and nanoscale size of AgNPs, which were suitable for the subsequent investigations.

### Light-dependent bactericidal effect of AgNPs.

**FIG 1:**
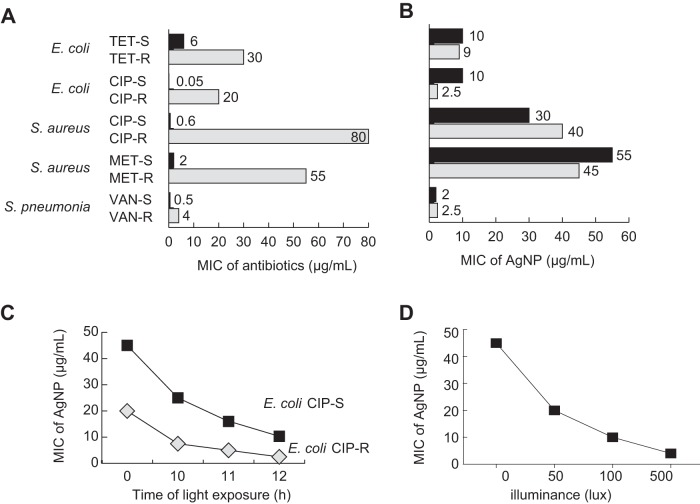
The antibacterial activity of AgNPs. (A and B) (A) MIC of antibiotics (left panel) and (B) AgNPs (right panel) for various sensitive (S) and resistant (R) bacteria. Abbreviations: TET, tetracycline; CIP, ciprofloxacin; MET, methicillin; VAN, vancomycin. The sensitive strains included E. coli BW25113, S. aureus ATCC 29113, and S. pneumoniae D39. (C) MIC of AgNPs for CIP-sensitive and -resistant E. coli strains after 0, 10, 11, and 12 h of light exposure. All MIC results were determined with a microdilution method in three independent biological replicates. (D) MIC of AgNPs for E. coli strains exposed to 0, 50, 100, and 500 lx of light.

To test the antibacterial activity of AgNPs, a series of antibiotic-sensitive and -resistant bacteria were used in this study, including E. coli, S. aureus, and S. pneumoniae (Fig. 1A). Impressively, AgNPs exhibited lower MICs for the resistant bacteria than for the wild-type bacteria in most cases, regardless of the type of resistance and species (Fig. 1B), under conditions of the normal room illumination of approximately 116.37 lx.

Silver is known for its light sensitivity: the Daguerreotype process required silver and its halides to obtain positive photographic prints. Therefore, we hypothesized that light exposure might promote stronger bactericidal activity of AgNPs due to light excitation. To verify this hypothesis, the MIC values of AgNPs against E. coli BW25113 under conditions of different durations of light exposure were determined. Consistent with our hypothesis, longer light exposure remarkably lowered the MICs of AgNPs for both ciprofloxacin (CIP)-sensitive and CIP-resistant E. coli (Fig. 1C), demonstrating stronger inhibitory activity.

To further determine the relationship between light exposure and the MIC of AgNPs, white light with different intensities of 0 to 500 lx was used to irradiate bacteria in the presence of AgNPs. MIC values decreased with increased illumination, suggesting that increased light intensity enhanced the antibacterial effect of AgNPs (Fig. 1D).

White light behaved as polychromatic light. Next, monochromatic light (blue, purple, red, and yellow light) at the same intensity as the white light (∼116.37 lx) was also used to activate the bactericidal activity of AgNPs in this study. Blue light promoted the bactericidal activity of AgNPs more effectively than the other colors. Considering that the typical room light excitation (116.37 lx) was already effective, 116.37 lx white light was selected for use in subsequent experiments to represent the clinical environment.

### The bactericidal effect is independent of Ag^+^ and ROS.

A previous study posited that Ag^+^ released by AgNPs is a major antibacterial substance (14). The levels of oxidization and release of the Ag^+^ ions from AgNPs have been found to be strongly dependent on the oxygen content of the media (27). Under physiological conditions, strong oxidizing agents such as concentrated nitric acid are not present, and therefore the only energy that could ionize silver is that of photons. The first ionizing potential of silver is 7.576 eV, which requires deep-UV photons with a maximum wavelength of 163 nm to ionize silver as Ag^+^. However, 163-nm-wavelength deep-UV light is absorbed by ozone and thus cannot penetrate the atmosphere, making it extremely rare in nature unless artificially generated by a deep-UV light source. In contrast, the 404-nm-wavelength absorption peak in the visible spectrum of AgNPs indicated that much lower energy could be absorbed by the AgNPs (data not shown). Therefore, we hypothesized that the AgNPs did not release Ag^+^ when inhibiting bacteria.

**FIG 2:**
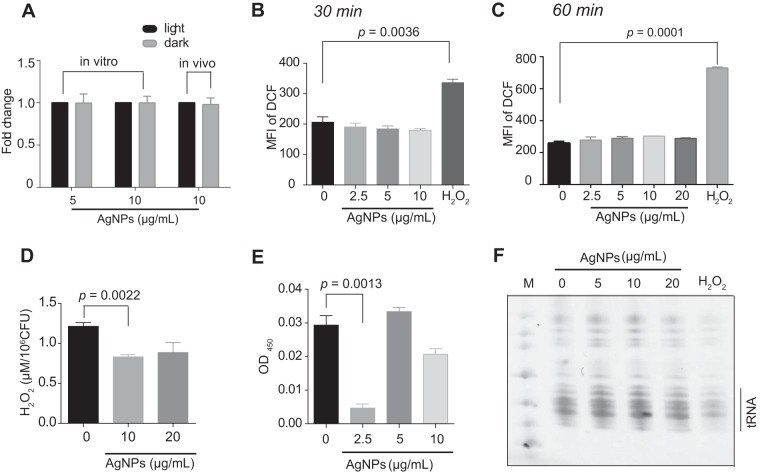
The bactericidal effect of AgNPs is independent of Ag^+^ and ROS. (A) Ag^+^ concentration released from AgNPs after incubation in LB medium and in cells for 12 h in dark or light conditions. (B and C) Intracellular ROS concentration after 30 min (B) and 60 min (C) of AgNP treatment under light. The cells treated with 2 mM H_2_O_2_ were used as a positive control. MFI, mean fluorescence intensity. (D) Extracellular H_2_O_2_ concentration after AgNP treatment under light for 30 min. (E) Intracellular O_2_**^.–^** concentration after AgNP treatment under light for 30 min. (F) tRNAs in E. coli treated with AgNPs of different concentrations and 15 min of light, resolved using PAGE. E. coli treated with 5 mM H_2_O_2_ was used as a positive control. Data are represented as means ± SEM. *, *P < *0.05; **, *P* < 0.01; ***, *P < *0.001.

To test this hypothesis, we measured the concentration of Ag^+^ released from AgNPs both in Luria-Bertani (LB) medium and in cells in a filter unit with or without natural light exposure using inductively coupled plasma mass spectrometry (ICP-MS). The results revealed no differences in the levels of Ag^+^ released from AgNPs in both the medium and the cells in the presence or absence of light (*P > *0.99, two-tailed *t* test) (Fig. 2A), which validated our hypothesis. Therefore, the light-induced antibacterial activity of AgNPs is independent of Ag^+^.

Previous literature has proposed that ROS might represent a possible antibacterial mechanism of AgNPs (28–31). Therefore, we tested the ROS in E. coli with and without AgNP treatment under light. The ROS level was not increased in E. coli after AgNP treatment for 15 min and 30 min (Fig. 2B and C). Furthermore, the H_2_O_2_ and O_2_**^.–^** contents in E. coli after AgNP treatment were also not increased under light (Fig. 2D and E). It has been reported that the tRNA level is globally decreased in bacteria upon oxidative stress for 15 to 30 min to decelerate translation elongation for survival (32). However, the tRNA level seen in cells upon AgNP treatment was similar to that in untreated cells, while the H_2_O_2_-treated cells showed a considerable tRNA decrease (Fig. 2F). These results confirmed that oxidative stress is not the major bactericidal mechanism of AgNPs.

### AgNPs transfer light energy to proteins and catalyze protein aggregation.

**FIG 3:**
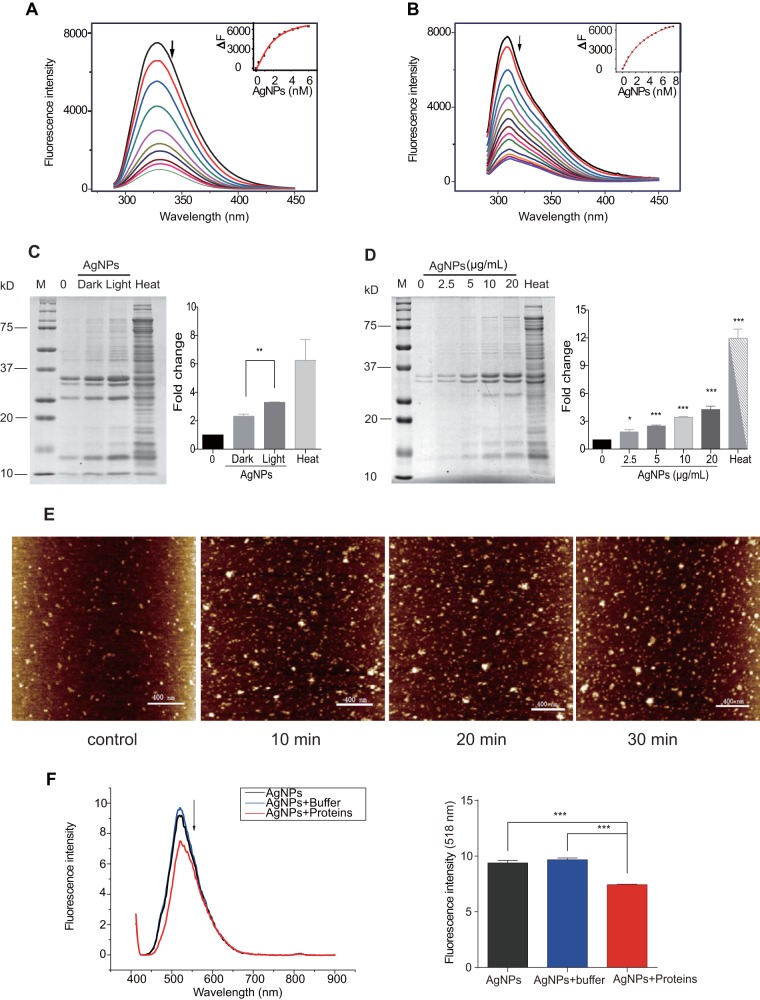
AgNPs catalyze protein aggregation by transferring light energy to proteins. (A and B) Fluorescence titration spectra of GAPDH (A) and Gmk (B) with 280-nm-wavelength excitation. Aliquots of AgNPs were added to GAPDH (3 μM) and Gmk (2 μM), respectively. The fluorescence quenching (ΔF) at 329 nm versus AgNP concentration was fitted with the Hill equation. (C) Detergent-insoluble proteins (DIPs) of E. coli under conditions of AgNP treatment with light or in the dark. (D) Dependence of E. coli DIPs on AgNP concentration. AgNPs (∼2.5 to ∼20 μg/ml) were added into the E. coli culture under light. Heat treatment was conducted as a positive control for protein aggregation. The relative intensity of the entire lane is plotted in the right panel. (E) AFM images of protein aggregation with AgNP treatment. Total cell proteins (1 mg/ml) were treated with 10 μg/ml AgNPs for 0, 10, 20, and 30 min. (F) Fluorescence spectra of AgNPs excited at 404 nm (left panel). E. coli proteins (32 μg) were added to the AgNP (500 μg/ml) solution, and fluorescence values at 518 nm were recorded (right panel). Results were analyzed using a two-tailed unpaired *t* test and three repeats. Error bars represent means ± SEM. *, *P < *0.05; **, *P* < 0.01; ***, *P < *0.001.

Since AgNPs do not release Ag^+^ or produce ROS, the particles must perturb proteins by direct contact, i.e., representing a “bind-and-damage” model. We measured the binding of AgNPs with two common bacterial proteins, glyceraldehyde-3-phosphate dehydrogenase (GAPDH) and guanylate kinase (Gmk), using fluorescence quenching titration at 329 nm and 309 nm, respectively. The Hill equation-fit titration curves revealed association constant (*K*_a_) values of 5.7 × 10^8^ M^−1^ and 2.4 × 10^8^ M^−1^ for GAPDH and Gmk, respectively (Fig. 3A and B), indicating a universal binding affinity of AgNPs for various kinds of proteins. The fluorescence quenching indicated a remarkable structure alteration of the proteins, suggesting that the AgNPs may induce protein misfolding and aggregation.

To further test this postulation, we extracted detergent-insoluble protein (DIP) aggregates from AgNP-treated E. coli with or without light exposure. The E. coli cultured at 42°C served as a positive control for massive protein aggregation. Although 10 μg/ml AgNPs in darkness and light without AgNPs induced slight protein aggregation, light-excited AgNPs induced much stronger and global protein aggregation (Fig. 3C). We also found that the protein aggregation increased in a dose-dependent manner (Fig. 3D). Subsequently, protein aggregation was monitored by atomic force microscopy (AFM). We found that AgNP-treated proteins formed aggregates quickly (in 10 min) (Fig. 3E). Experiments showed that AgNP treatment in the presence of light resulted in an increase in both spot number and area of protein aggregates. Since light is essential for efficient protein damage, we presumed that light-induced protein oxidization (33) might be the major pathway for bacterial inhibition, and AgNPs served as a catalyst. These results validated our bind-and-damage model. Accumulating damaged proteins and aggregates would induce cytotoxicity, which could explain the bactericidal activity of the AgNPs.

Since protein aggregation requires energy (34, 35), we then hypothesized that the protein structure alteration was caused by transferal of light energy to the proteins by AgNPs. Under conditions of excitation by 404-nm-wavelength violet light, AgNPs released the absorbed light energy in the form of 404-nm-wavelength emission in the absence of proteins (Fig. 3F). This emission light was quenched in the presence of proteins (Fig. 3F), indicating that the absorbed light energy was transferred to proteins, causing the damage to protein structures.

### Light-excited AgNPs circumvent bacterial protection mechanisms.

**FIG 4:**
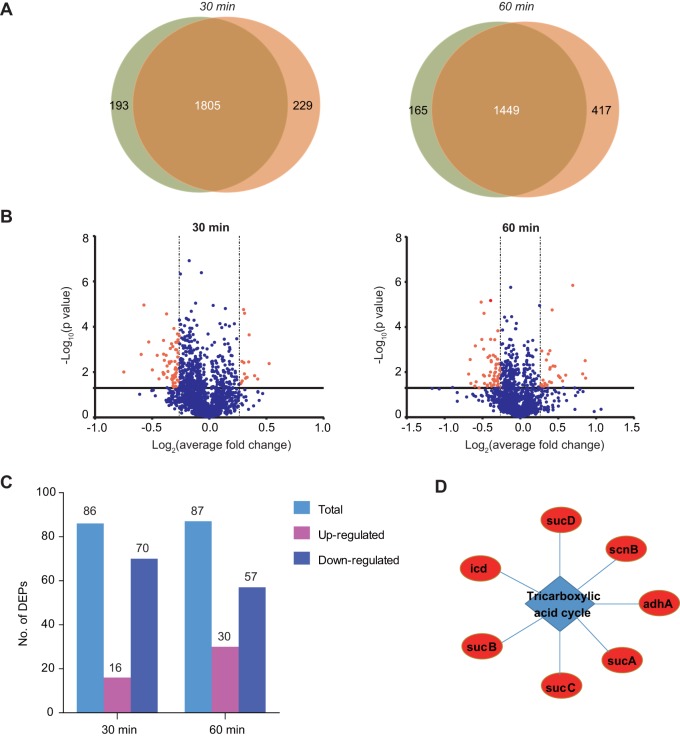
Proteomic analysis of E. coli response to AgNP treatment under conditions of light and of dark. (A) Venn diagrams of the number of proteins quantified using quantitative proteomics. E. coli was treated with 5 μg/ml AgNPs for 30 min and 1 h in two biological repetitions, respectively. (B) Volcano plot of the differentially expressed proteins (DEPs) at the two time points. (C) Numbers of DEPs in the 30-min and 1-h AgNP treatments under light compared with those in the dark. (D) GO enrichment (biological process) of the upregulated proteins in 1-h AgNP treatment under light. The tricarboxylic acid cycle was enriched, with *P = *2.76 × 10^−8^.

With such stress from AgNPs, bacteria may respond with various stress-response systems for survival. To depict the global response to the AgNP treatment, we performed isobaric tags for relative and absolute quantitation (iTRAQ)-based proteomics to quantify the differentially expressed proteins (DEPs) of E. coli upon 5 μg/ml AgNP treatment for 30 min or 1 h in the presence versus absence of light. We identified 1,805 and 1,449 proteins from the two time points, respectively, showing concordance (Fig. 4A). DEP analysis revealed 16 and 70 upregulated and downregulated proteins at 30 min, respectively (Fig. 4B, left panel), and 30 and 57 upregulated and downregulated proteins at 1 h, respectively (Fig. 4B, right panel).

As a classical response to massive protein aggregation, the levels of the chaperones should have been elevated. Surprisingly, none of the chaperones (GroEL/ES, DnaK/J/E, ClpP, etc.) were upregulated at 30 min of AgNP treatment under light, and only GroES was found to be marginally (1.20-fold) upregulated after 1 h. The system that responds to oxidative stress, e.g., OxyR and SoxRS, was also not upregulated at any time points, suggesting that this system did not react. This was echoed by the constant or even increasing tRNA levels (Fig. 2F). The only enriched GO term (*P < *0.001) of the upregulated proteins at 30 min was the translation process (*P = *5.83 × 10^−5^), which includes solely a series of ribosomal proteins, indicating that the bacteria attempted to produce more ribosomes when treated with AgNPs under light. This result suggested that the stringent response was not induced, since the stringent response would suppress translation to conserve energy for survival. In sum, the AgNPs circumvented all the stress-response systems of bacteria, indicating that it may be very difficult for the bacteria to develop effective resistance against the light-excited AgNPs.

In another aspect, the DEPs against light-excited AgNPs at 1 h were highly enriched (*P < *0.001) in only one pathway, i.e., the tricarboxylic acid cycle pathway (*P = *2.76 × 10^−8^) (Fig. 4D). This pathway could not specifically counteract any nutrient-independent stress, suggesting that the bacteria could not efficiently counteract the light-excited AgNPs.

### AgNPs inhibit pan-drug-resistant bacteria with NDM-1.

Since AgNPs can circumvent bacterial defense systems, we hypothesized that the AgNPs can also effectively inhibit pan-drug-resistant bacteria with the carbapenemase NDM-1. We chose an extremely pan-drug-resistant bacterial strain, Acinetobacterbaumannii ABC3229, which was isolated from a patient and contains an NDM-1 gene, which encodes carbapenemase. Moreover, this strain contains β-lactamase gene *tem-1* and 16S rRNA methylase gene *armA*, which endow to the bacteria an even wider resistance spectrum. Antibiotic susceptibility tests revealed that this strain is resistant to imipenem, meropenem, cefepime, cefotaxime, ceftazidime, aztreonam, ampicillin/sulbactam, cefoperazone/sulbactam, piperacillin-tazobactam, minocycline, tigecycline, gentamicin (GEN), amikacin, and CIP (36). The MICs of these antibiotics for this strain were at least 64× higher than those for the sensitive strains. In sharp contrast, the MIC of AgNPs for this superbug was 0.9 μg/ml, remarkably lower than the MICs of those antibiotics. Interestingly, the MIC of AgNPs for the sensitive A. baumannii ATCC 19606 strain was 2.1 μg/ml, more than 2-fold higher than that for the NDM-1-containing pan-drug-resistant strain. This indicated that the AgNPs are more effective on superbugs than sensitive bacteria.

## DISCUSSION

Numerous investigations of TiO_2_-embedded AgNPs as a photocatalyst for disinfection have been conducted (37, 38). Nevertheless, TiO_2_ itself is a photocatalyst that induces ROS (reviewed in reference 39) and can damage lipids and proteins under UVA light (40), and AgNPs could only enhance this effect. Here, we focused on the bactericidal effect of pure AgNPs and identified a new antibacterial mechanism distinct from that of ROS-inducing TiO_2_. AgNPs can penetrate the cell wall and membrane and then gather in the cytosol (8, 20, 41), allowing direct binding to cytosolic proteins and induction of intracellular protein aggregation. This mechanism is far more effective than that of TiO_2_ and TiO_2_-embedded AgNPs, which work only outside the bacteria.

The photocatalytic properties of AgNPs, especially the release of Ag^+^ and the production of ROS, were acknowledged in reports from previous studies, but only in the context of UV irradiation (42, 43). The reason is clear: UV light could provide sufficient energy for the interband transition, exciting the ground-state electrons of the 4d band to the energy level where oxygen molecules can seize them, resulting in ROS. Visible light contains insufficient energy to achieve this effect (42). Our results confirmed that the AgNPs under visible light could not produce Ag^+^ or ROS but could still catalyze massive protein aggregation. Undoubtedly, visible light is much more practical than UV light for both industrial and medical applications.

The most exciting feature of AgNPs is that they circumvent all known bacterial resistance mechanisms. The oxidative stress-response system, stringent response, inhibition of protein synthesis, and heat shock response were not triggered by AgNPs. Interestingly, the chaperone systems that could facilitate protein folding were not activated, probably because of the unique unfolding process induced by the light-excited AgNPs. Therefore, the bacteria cannot prevent or reverse the accumulating protein aggregation, which may lead to significant cytotoxicity. This mechanism is also independent of the bacterial species and thus should be applicable to any bacteria. Indeed, we have shown that AgNPs are effective toward E. coli, S. aureus, S. pneumoniae, and A. baumannii. Moreover, the protein aggregation mechanism overwhelms any specific antibiotic resistance mechanisms such as antibiotic degradation enzymes, mutations of the drug targets, and channel protein overexpression—making AgNPs a promising potential “savior” against the current flood of antibiotic-resistant bacteria.

Another interesting feature of AgNPs is their higher efficacy against resistant bacteria than against sensitive bacteria in most cases (Fig. 1A; see also the case of NDM-positive pan-drug-resistant A. baumannii bacteria). This mechanism could be deduced from the proteomic response to AgNP treatment. After 1 h of AgNP treatment under light, the only upregulated pathway was the central carbon metabolism (Fig. 4D). As central carbon metabolism remarkably contributes to drug resistance (44, 45), these pathways are already highly upregulated in resistant bacteria; thus, further upregulation of them is more difficult.

**FIG 5:**
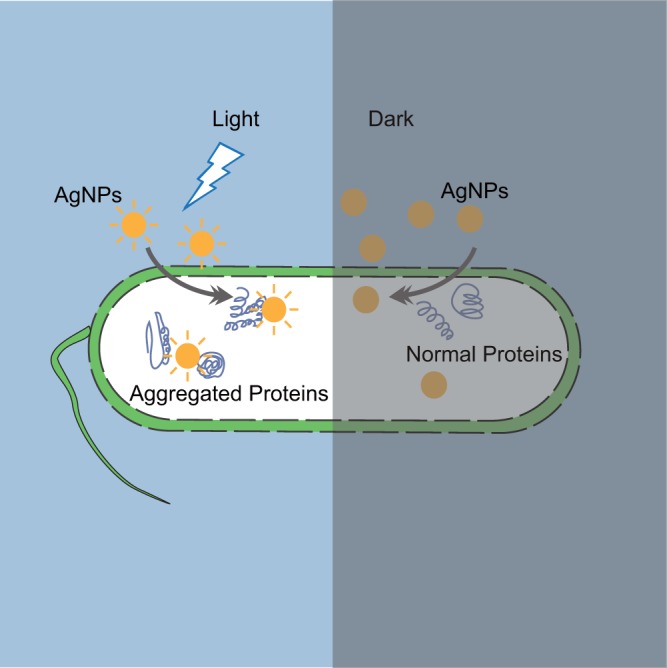
The bactericidal mechanism of AgNPs: photocatalytic induction of massive aggregation of cellular proteins under visible light.

In sum, we revealed the bactericidal mechanism of AgNPs: photocatalytic induction of massive aggregation of cellular proteins under visible light (Fig. 5). This effect could not be counteracted by known bacterial stress-response mechanisms. Therefore, AgNPs are a promising broad-spectrum bactericide, especially against antibiotic-resistant bacteria, with little risk of development of resistance.

## MATERIALS AND METHODS

### Bacteria and AgNPs.

E. coli K-12 BW25113 was purchased from Coli Genetic Stock Center (CGSC, New Haven, CT, USA). S. aureus ATCC 25923 and A.baumannii ATCC 19606 were purchased from the American Type Culture Collection (ATCC, Manassas, VA, USA). S. pneumoniae D39 strain NCTC 7466 was obtained from the National Collection of Type Culture (NCTC, London, United Kingdom). S. aureus ATCC 29213 was acquired from the Third Affiliated Hospital of Sun Yat-Sen University (Guangzhou, China). A. baumannii AB3229 containing NDM-1 plasmid and MDR-ZJ06 were acquired from Sir Run Run Shaw Hospital, Zhejiang University (Hangzhou, China). Tetracycline (TCY)-resistant and ciprofloxacin (CIP)-resistant E. coli K-12 BW25113, CIP-resistant S. aureus ATCC 29213, methicillin (MET)-resistant S. aureus ATCC 25923, vancomycin (VAN)-resistant S. pneumoniae D39, and CIP-resistant and gentamicin (GEN)-resistant A. baumannii were cultivated from the corresponding sensitive strains in our laboratory. AgNP (5 mg/ml) coated with polyvinylpyrrolidone (PVP) was purchased from nanoComposix (San Diego, CA, USA).

### Characterization of AgNPs.

Morphological examination of AgNPs was carried out by TEM (JEM-2100F, Tokyo, Japan) with an acceleration voltage of 80 kV. AgNP solution (10 μg/ml) was added to a copper grid and dried. DLS (Zetasizer Nano ZS, Malvern, United Kingdom) was used to analyze the size of AgNPs.

### Antibacterial activity of AgNPs.

The microdilution method (46) was used with minor modifications in this study to determine the MIC of AgNPs against antibiotic-sensitive and antibiotic-resistant E. coli, S. aureus, S. pneumoniae, and A. baumannii. For all experiments in this study, E. coli and A. baumannii were cultured in LB medium at 37°C with shaking at 200 rpm; S. aureus was cultured in tryptic soy broth (TSB) medium at 37°C with shaking at 200 rpm; and S. pneumoniae was cultured in Todd-Hewitt broth (Oxoid, Basingstoke, United Kingdom) with 0.5% yeast extract medium at 37°C in 5% CO_2_. Briefly, bacteria (2 × 10^6^ CFU/ml) were incubated with AgNPs of different concentrations for 12 h in a 48-well plate, and then the values corresponding to the optical density at 600 nm (OD_600_) were measured for each well using a microplate reader. The concentration of AgNPs resulting in no bacterial growth (OD_600_ < 0.05) was identified as the MIC.

### MICs of AgNPs and growth curve assays under different light conditions.

We used the microdilution method (46) to evaluate the MICs of AgNPs for E. coli BW25113 under light and dark conditions with minor changes. To determine the MIC of AgNPs without light exposure, all the experimental steps were conducted in absolute darkness and guided with infrared night vision (Pulsar EDGE GS1x20, Lida, Belarus). Overnight-cultured bacteria were diluted to 2 × 10^6^ CFU/ml with fresh medium. Then, three groups of bacteria were separately cultured for 2 h in darkness (10 h in light), 1 h in darkness (11 h in light), or 0 h in darkness (12 h in light) with AgNPs of different concentrations. The OD_600_ values of each plate were determined by the use of a microplate reader. The concentration of AgNPs resulting in an OD_600_ value under 0.05 was considered the MIC.

The spectrum of the white light used in the experiments was determined with a spectrometer (FLAME-T-VIS-NIR-ES; Ocean Optics, Largo, FL, USA). The light intensity was determined with a light meter.

To determine growth curves, E. coli BW25113 was cultured in fresh LB medium, grown to an OD_600_ of 1.0, diluted 1:100 with a series of AgNP concentrations in a 24-well plate, and cultured at 37°C for 8 h under different light sources. Bacterial densities were determined each hour at 600 nm with a microplate reader (ELx800; BioTek, Winooski, VT, USA). To avoid daylight interference, the bacteria were cultured in a dark room and then wrapped with tinfoil prior to measurement with the microplate reader. The measurement at each time point was performed with one 24-well plate, so each growth curve was determined based on eight plates. To determine the growth curves of bacteria under monochromatic light, blue, purple, red, and yellow light with the same intensity as white room light were used as light sources. All monochromatic lights were 9-W light-emitting-diode (LED) lamps of 0.6 m in length and were fixed on the top of the incubator, and the spectra were the same as those reported in a previous study (47).

### Detection of silver ions released from AgNPs.

The AgNP solution was added to LB medium at final concentrations of 5 μg/ml and 10 μg/ml and was then incubated overnight under light and dark conditions, separately. The diluted AgNP solution was filtered using a 3-kDa Millipore filter to remove AgNPs and washed twice with 1 ml MilliQ water. All eluates were combined to determine the concentration of Ag^+^.

To detect the silver content inside bacterial cells, 10 μg/ml AgNPs was added to E. coli BW25113 cells at an OD_600_ of ∼0.6 and subsequently cultured at 37°C and 200 rpm for 12 h under light and dark conditions, separately. After culture, cells were collected by centrifugation at 12,000 × *g*, washed three times with MilliQ water, resuspended with 500 μl of MilliQ water, and then lysed with sonication with a 20% pulse (3 s on, 3 s off) for 15 min. The cell debris was removed by centrifugation at 12,000 × *g* for 30 min, and the supernatant was carefully collected.

All eluted samples were used to determine the concentration of silver ions by ICP-MS (Optima 2000 DV; PerkinElmer, USA) according to a previously described method (48). The concentrations of Ag^+^ released from AgNPs under light and dark conditions were compared.

### ROS content detection.

We detected the ROS content of E. coli BW25113 treated with AgNPs for 15 min, 30 min, and 1 h by fluorescent probe using 2′,7′-dichlorofluorescein diacetate (DCFH-DA) as described in a previous report (34). H_2_O_2_-treated E. coli cells were used as a positive control. Briefly, DCFH-DA was added to the E. coli culture once it reached an OD_600_ of ∼0.5 and incubated for 30 min. The E. coli cells were washed twice with phosphate-buffered saline (PBS) buffer. Then, 0, 2.5, 5, 10, and 20 μg/ml AgNPs and 2 mM H_2_O_2_ were used to treat the E. coli cells for 1 h. Subsequently, the bacteria were washed with PBS. The fluorescence intensities were determined by flow cytometry (C6, BD Biosciences, San Jose, CA, USA).

### Quantitation of H_2_O_2_.

The H_2_O_2_ content of E. coli BW25113 treated with 10 μg/ml or 20 μg/ml AgNPs for 30 min was detected with an Amplex Red hydrogen peroxide/peroxidase assay kit (Invitrogen, Carlsbad, CA, USA) according to the method used in our previous study (49). Briefly, E. coli BW25113 cells were collected at an OD_600_ of ∼0.5 and centrifuged at 8,000 × *g* to collect the supernatants, which were filtered with 0.22-μm-pore-size filters and then incubated with H_2_O_2_ working solution. Finally, the H_2_O_2_ content in E. coli cells that had been left untreated or treated with AgNPs was measured by a microplate reader with excitation at 530 to 560 nm and emission at 590 nm.

### Quantitation of O_2_^.–^.

The O_2_^.–^ content in E. coli BW25113 was detected with a superoxide assay kit (Beyotime, Haimen, China) based on a previously described method (35). In brief, the bacteria subjected to different treatments were collected at an OD_600_ of ∼0.5 by centrifugation and washed with PBS. Then, the cell pellets were resuspended to 10^6^ CFU/ml with the superoxide working buffer according to the product manual. Next, the bacteria were incubated in 48-well microplates for 3 min and then treated with 0, 2.5, 5, or 10 μg/ml AgNPs for 30 min. Finally, the absorbance at 450 nm was recorded by the use of a microplate reader, with *A*_630_ as the reference wavelength.

### tRNA level detection.

When E. coli BW25113 had grown to an OD_600_ of ∼0.5, the cultures were treated with 5, 10, or 20 μg/ml AgNPs and 2 mM H_2_O_2_. After 15 min of treatment, total RNA of E. coli BW25113 was extracted using the TRIzol method as described in a previous report (32). Then, 200 μg total RNA was loaded on a polyacrylamide gel. Bands corresponding to ∼70 to∼100 bp were considered representative of tRNA (32). The band intensities of total tRNA were quantitated using ImageJ (1.50i; National Institutes of Health, Bethesda, MD, USA).

### Fluorescence spectroscopy.

The binding abilities of AgNPs with GAPDH and Gmk protein were investigated with a fluorescence spectrometer (model F7000; Hitachi, Tokyo, Japan) according to a previously described method (48). The parameters were set as follows: excitation wavelength at 280 nm (slit width of 5.0 nm) and emission wavelength between 290 nm and 450 nm (slit width of 5.0 nm). AgNPs were added dropwise to 1.5 ml GAPDH or Gmk protein solution (3 μM protein, 20 mM Tris-HCl, 100 mM NaCl, pH 7.4). Fluorescence spectra were measured after each titration until the spectra were stable. The relative changes in fluorescence emission (ΔF) at 329 nm and 309 nm during the titration versus the concentrations were fitted to titration curves. The data were analyzed with the Hill plot equation by Origin 7.5 to obtain association constants (*K*_a_). The concentration of AgNPs was transferred by using the density of silver (10.5 g/cm^3^ at 27°C) (14), and the molar mass was calculated by the following equation:

$$M=N_{A}\cdot m_{\text{AgNP}}=N_{A}\cdot \rho_{\text{Ag}}\cdot \frac{4}{3}\pi {\left(\frac{d}{2}\right)}^{3}$$

where *N_A_* is *N* Avogadro constant, *m*_AgNP_ is the mass of a AgNP sphere, and ρ_Ag_ is density of Ag.The molar concentration was further calculated based on the obtained molar mass. The molar concentration of 5 mg/ml AgNP solutions is 1.1 μM. Then, 300 nM concentrations of the AgNPs used in the titration were diluted from 5 mg/ml AgNP solutions with MilliQ water.

### Extraction of detergent-insoluble proteins (DIPs).

E. coli BW25113 at an OD_600_ of ∼0.5 was treated with 10 μg/ml AgNPs under light and in the dark for 1 h separately. Meanwhile, E. coli BW25113 heated at 48°C for 20 min was used as a positive control. Furthermore, in order to compare DIP changes in E. coli under conditions of AgNP treatment with different concentrations, we treated E. coli with 0, 2.5, 5, 10, and 20 μg/ml AgNPs for 1 h. Then, the DIPs were extracted using a previously described method (50).

### iTRAQ-based proteomic analysis.

Total proteins were extracted from E. coli BW25113 cultivated to the early log phase and then treated with 5 μg/ml AgNPs under light and in the dark for 30 min and 1 h, respectively. The bacteria were centrifuged at 8,000 × *g* for 30 min, and the cell pellets were washed with PBS three times. Subsequently, the collected cells were lysed using SDS lysis buffer. A bicinchoninic acid (BCA) protein assay kit (Thermo Fisher Scientific) was employed to determine protein concentration. The protein samples were digested with trypsin, and the collected peptides (150 μg) of each group were labeled with three different iTRAQ reagents. The labeled peptides were mixed and separated into six groups according to polarity using reverse-phase ultra-high-pressure liquid chromatography (UPLC) with an Ultremex SCX column (Phenomenex, Torrance, CA, USA) (4.6 mm by 250 mm, 5-μm pore size). The collected fractions were dried and resolved with buffer A (5% acetonitrile and 0.1% formic acid). A 4-μl volume of each fraction containing about 0.5 μg/ml peptides was subjected to mass spectrometry using an Orbitrap Fusion Lumos system (Thermo Fisher Scientific) with previously described parameters (46). The raw data were searched against the E. coli K-12 database by the use of Protein Discoverer software. The parameters were set as follows: sample type, iTRAQ 4plex (peptide labeled); Cys alkylation, iodoacetic acid; digestion, trypsin; database, E. coli K-12.fasta; unique peptides, ≥1, false-discovery rate (FDR), <0.01.

The proteins identified in two repeated experiments were used for quantitation. The proteins with fold change values above 1.2 or below 0.83 were considered DEPs. GO enrichment of the DEPs was performed using the DAVID website (https://david.ncifcrf.gov/).

### AFM detection of protein aggregation.

Whole protein was extracted from E. coli BW25113 using the method mentioned above and filtered with a 100-kDa ultrafilter to remove cell debris. The collected proteins (1 mg/ml) were treated with 1 μg/ml AgNPs at 37°C for 10, 20, and 30 min. After the reaction, the protein solutions were redissolved in double-distilled water (ddH_2_O) to reach a final concentration of 10 μg/ml, and 5 μl of each sample was deposited onto a newly caved mica surface at room temperature. Images were acquired with an atomic force microscope (MultiMode NanoScope-V; Veeco Instruments, Plainview, NY, USA). The scan rate was 1 Hz, the feedback value was 5.289, and ScanAsyst Auto Control was used. NanoScope Analysis software was used for data processing.

### Statistical analysis.

Statistical analysis was carried out using the two-tailed unpaired Student's *t* test with GraphPad Prism version 5 (La Jolla, CA, USA). Data are presented as means ± standard errors of the means (SEM) of results from at least three biological replicates. *P* values of <0.05 were considered significant for all experiments.

### Data availability.

The raw proteomics data and search results have been deposited in the ProteomeXchange Consortium via the PRIDE (51) partner repository with the data set identifier PXD009674 and can be accessed with the reviewer account (website, http://www.ebi.ac.uk/pride; user name, reviewer76801@ebi.ac.uk; password, R4VrXvDu).
